# A 3-Bit Low-Profile High-Gain Transmissive Intelligent Surface for Beam Focusing and Steering Applications

**DOI:** 10.3390/mi16121399

**Published:** 2025-12-12

**Authors:** Zaed S. A. Abdulwali, Majeed A. S. Alkanhal

**Affiliations:** Electrical Engineering Department, King Saud University, Riyadh 11421, Saudi Arabia

**Keywords:** unit cell, varactor diode, beam steering, configurable lens, transmissive intelligent surfaces, metasurfaces, transmittarray

## Abstract

This paper presents a 3-bit transmissive intelligent surface (TIS) using a novel technique that employs a unit cell comprising loaded semi-loop dipole resonators. The two resonators are anti-symmetrically oriented along the H-plane, functioning as transmitter and receiver on opposite sides of the TIS. The unit cell, with 13.2 mm periodicity, achieves 360° phase variation in 45° steps while maintaining insertion loss below 2 dB at 10 GHz. A 17 × 17 array TIS is designed using ray tracing and phase shift compensation techniques, with phase profiles distributed across eight discrete varactor states. The implemented TIS demonstrates a 10.8 dB gain enhancement for a horn antenna source at 10 GHz while preserving antenna matching, polarization, and radiation efficiency. The design achieves beam steering capabilities up to 60° with ±2° precision across elevation, azimuth, and inclined angles, maintaining an average steering gain loss of 3 dB over a 400 MHz bandwidth. These characteristics make the proposed design particularly effective for modern wireless coverage extension and tracking applications.

## 1. Introduction

**Figure 1:**
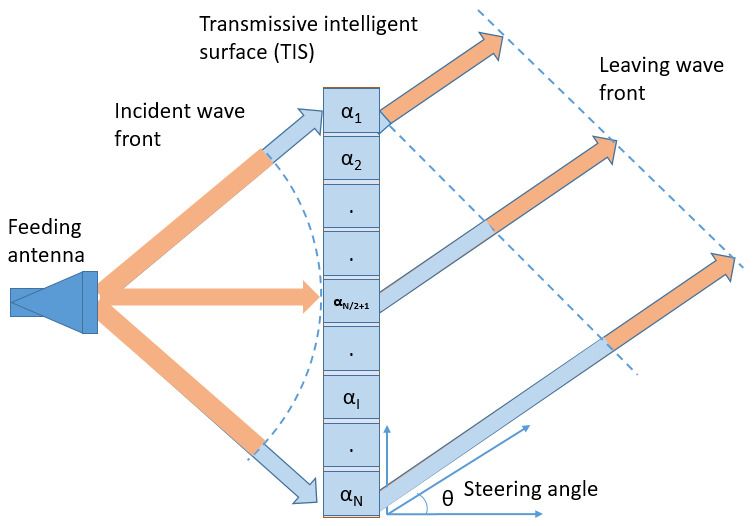
Schematic view of a function of a reconfigurable transmissive metasurface.

Antenna beam steering techniques have garnered significant attention for their potential to enhance wireless communication and tracking capabilities. Metasurfaces have emerged as a particularly promising solution, offering distinct advantages over conventional approaches such as dielectric lenses and phased array systems. These advantages include lightweight construction, flexibility, cost-effectiveness, and reduced power consumption [1,2,3,4,5]. Metasurfaces are composed of multilayer passive transmissive unit cells, strategically positioned at an optimal distance from the source [4,6,7,8]. Each unit cell functions as a signal processor, intercepting incoming electromagnetic waves and re-radiating them with controlled phase shifts while preserving signal amplitude. This mechanism enables precise beam focusing and redirection. The feeding source can be either a single antenna or an array of antennas integrated with a transmissive intelligent surface (TIS), creating an effective steering transmitarray antenna. Unlike reflectarray antennas that suffer from feed blockage, transmitarrays eliminate this limitation, allowing the integration of multiple sources or antenna arrays. This flexibility in source configuration makes the system adaptable in both size and performance [4,6,7]. For only beam focusing applications, passive elements of different phase characteristics are used to build a transmitarray [1,9,10]. However, for beam steering applications, the unit cells of TIS require phase reconfigurability to compensate for varying path lengths between the antenna and individual cells. As shown in Figure 1, spherical waves from the antenna reach different unit cells with varying phases due to unequal distances from the antenna’s phase center. Additionally, waves must maintain phase coherence in the plane perpendicular to the steering direction, despite traversing different distances. Without adjustable phase control (i.e., variable α), TIS cannot steer the beam of the source antenna.

Consequently, unit cell designs require controllable elements for phase manipulation, with options including active components such as a Varactor diode [3,5,11], PIN diode [12], and MEMS switches [13] or tunable materials such as liquid metal [14], and graphene [15]. For microwave applications in C-band to Ka-band, active components are usually used. Varactor and PIN diodes exhibit higher ohmic losses than MEMS switches, but they offer superior switching speeds and reliability [6,7]. Varactor diodes are particularly advantageous, as their voltage-controlled variable capacitance enables continuous and wide phase variations, delivering more precise beam steering compared to PIN diodes’ binary states [4,16]. The unit cell performance is determined by factors such as the range of phase variation, the level of insertion loss, the bandwidth of phase variation and insertion loss, and the cell profile. These factors need to be improved to achieve a high efficiency TIS for the required bandwidth. Researchers in [17] have presented a limitation of the obtained phase shift versus insertion loss and number of layers, requiring multiple layers for a full-scale phase shifter (i.e., greater than 360°). For example, in [5,18,19,20] multiple layers are used to design the TIS, where the varactor diodes are included to control the phase. However, an increasing number of layers reduces the radiation efficiency due to the insertion loss of each layer, resulting in low gain and aperture efficiency. Ongoing research on designing TIS is oriented to improve bandwidth [21,22], scanning range [3,11,23], gain and efficiency [2], and the unit cell profile [2,24]. However, wide scanning angle and efficient beam steering require optimized phase profiles and angularly stable unit cell responses [25,26,27]. Researchers used different techniques to design TIS, such as transmitting and receiving layers [5,16], frequency-selective surfaces (FSS) [3], or Huygens metasurfaces [28]. Unit cells of 360° phase variations are presented in [5,16] but the size and complexity increase when four substrates are used. In [28], a 2-D beam steering TIS, utilizing dual-layer Huygens elements, is presented with 18.4 dBi gain and 20% aperture efficiency at 13 GHz. A low-profile TIS with 3-bit phase compensation at Ku-band achieves 21 dBi gain and 30° beam steering. Researchers in [29] proposed a 1-bit Ku-band TIS using subwavelength H-shaped coupling slots with ±50° scanning and 17 dBi gain, but the TIS has a low aperture efficiency of 14%. As the cost and power loss concern for designing transmitter systems, TIS that has a wide bandwidth, high gain, high aperture efficiency, and low profile is always preferred. Therefore, it is important to obtain a TIS system with a low profile, high steering resolution, high aperture and radiation efficiencies, and high directivity with the best trade-off between the complexity of the control and TIS performance. This needs a unit cell design that has a low profile and wide phase variation with low insertion loss.

In this research, a loaded semi-looped dipole technique is introduced to design a reconfigurable unit cell with 3-bit phase quantization for the 10 GHz frequency band. The proposed unit cell has wide phase variations and low insertion loss. The transmit and receive elements of the unit cell are etched on two substrates, where their alignment is optimized to control the coupling and resonance effect. The effect of the incident angle on the unit cell performance is presented. The cell results were used to design a low-profile, high-gain, and high-aperture-efficiency 17 × 17 TIS that was configured to perform focusing and steering functionalities.

## 2. TIS Design and Configuration

### 2.1. Unit Cell Design

**Figure 2:**
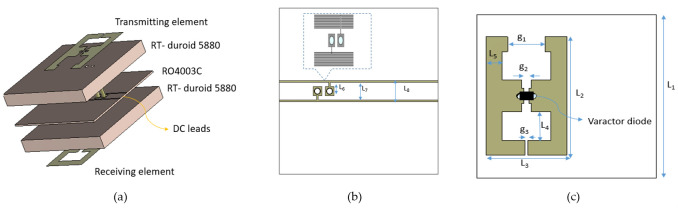
The proposed unit cell design and parameters (**a**) layers of the transmissive unit cell (**b**) DC feeding lines (**c**) resonance element.

Controlling the radiated beam of TIS is achieved by controlling the phase of the reradiated signal using TIS unit cells that have a transmission coefficient of low insertion loss and a wide phase variation response. To fulfill these requirements, a unit cell that relies on a looped dipole resonance is proposed. Initially, the low-loss dielectric substrate (i.e., Roger RT-duroid 5880 of dielectric constant of 2.2 and tangent loss of 0.0009) was chosen as a substrate for the resonant element due to its low loss. The substrate length and width are chosen to be around half of the wavelength of the central frequency of 10 GHz. A center-loaded semi-looped dipole resonance is proposed as a resonator, while the dimensions of the loop and dipole were initially tuned to a full and a quarter of the wavelength, respectively. The same resonator is used for both transmitter and receiver layers with asymmetric orientation, having a varactor diode as a centered load. The etched resonators on the opposite sides of the metasurfaces were decoupled or misaligned by a variable distance to control the total resonance. The layers of the proposed unit cell are shown in Figure 2a, where a middle thin layer is used for the DC biasing network and to separate the two main layers. The DC bias network is printed on Roger RO3004 of dielectric constant 3.55 and tangent loss of 0.0027, where the DC lines are collected together as a bus of parallel narrow lines symmetric around the *y*-axis and behind the central patch to not affect the RF performance. There is a via at each end of the varactor diode to connect it to the DC bias lines, as shown in Figure 2b. The Varactor diode is used to continuously control the phase of the resonance, as the design parameters were optimized to tune the unit cell for low insertion loss and wide phase variation. Therefore, low-loss Varactor diodes of type MAVR-000120-14110P from MACOM are chosen for both resonant elements, as shown in Figure 2c. The Varactor has a small average resistance (R) of 0.88 Ω, inductance (L) of 0.02 nH, and controllable capacitance (C) values [30]. As the biasing voltage varies from 0 V to 15 V, the capacitance varies continuously from 0.1 pF to 1 pF, which matches the required capacitance variation for our proposed resonator. The final optimized dimensions of the unit cell are listed in Table 1 in millimeters.

### 2.2. Results of the Unit Cell

**Figure 3:**
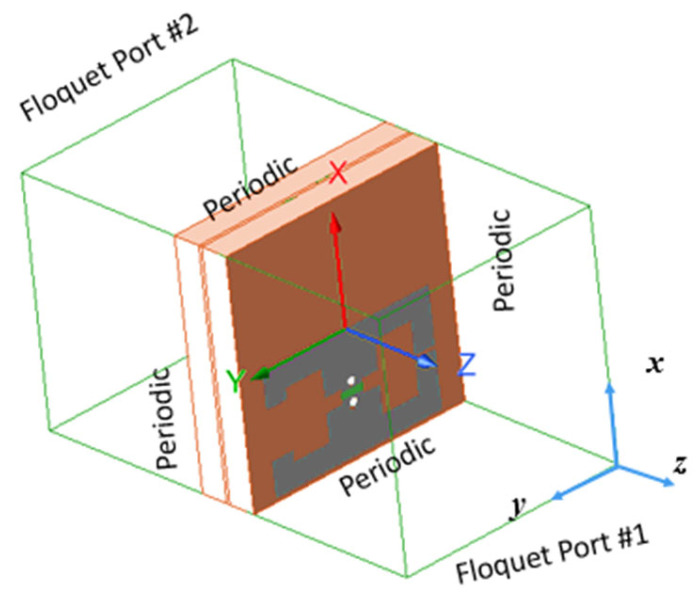
Proposed unit cell simulation setup.

The unit cell is simulated in the full-wave simulator (i.e., CST Microwave Studio) with periodic boundary conditions under plane wave excitations using two Floquet ports as shown in Figure 3. This simulation method is used to simulate and characterize frequency-selective surfaces (FSS) and metasurfaces in CST Microwave Studio. It considers the scattering problem as an infinite homogeneous surface or an infinite planar array of unit cells. This enables including the mutual coupling effect among surface unit cells. The excitation signal is a horizontally polarized plane wave, which incident normally on the unit cell as shown in Figure 3. The lumped load is used to represent the equivalent circuit for the Varactor diode that is put between the centered dipoles. The Varactor diode is modeled in the full-wave simulator as an RLC series equivalent circuit with values equal to those given by the manufacturer.

**Figure 4:**
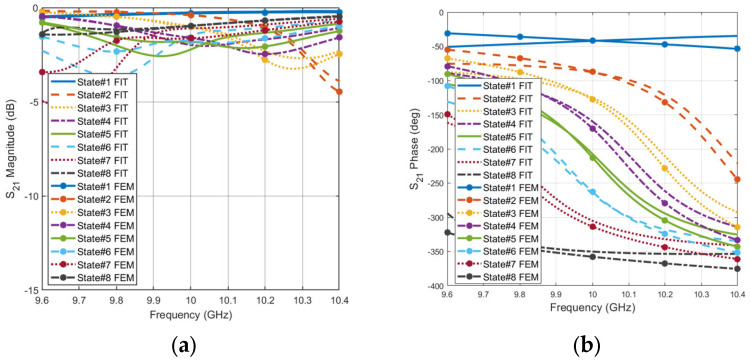
Unit cell transmission coefficient for all phase states (**a**) Magnitude (**b**) Phase.

The transmission coefficient (S_21_) parameters are calculated for the eight phase states. To validate the unit cell performance, two different full-wave solvers are used for simulating the same unit cell structure. The finite element method (FEM) of frequency domain solver and the finite integration technique (FIT) of time domain solver in CST Microwave Studio. The varactor capacitances of the equivalent circuit are set to [0.1 pF, 0.23 pF, 0.27 pF, 0.30 pF, 0.33 pF, 0.36 pF, 0.43 PF, 1pF] for states: {State #1, State #2, State #3, State #4, State #5, State #6, State #7, State #8}, respectively. Figure 4 shows the variation in amplitude and phase of S_21_ with the different values of capacitance. There are eight states for the unit cell response depending on the value of the capacitance. At the center frequency of 10 GHz, the amplitude values of the eight states are less than 2 dB, ensuring the low insertion loss for the proposed unit cell. The insertion loss is the total loss due to the dielectric, metallic, ohmic, and varactor losses. The good agreements between both FEM and TIS solvers validate the performance of the proposed semi-loop dipole resonators loaded with Varactor diodes.

**Figure 5:**
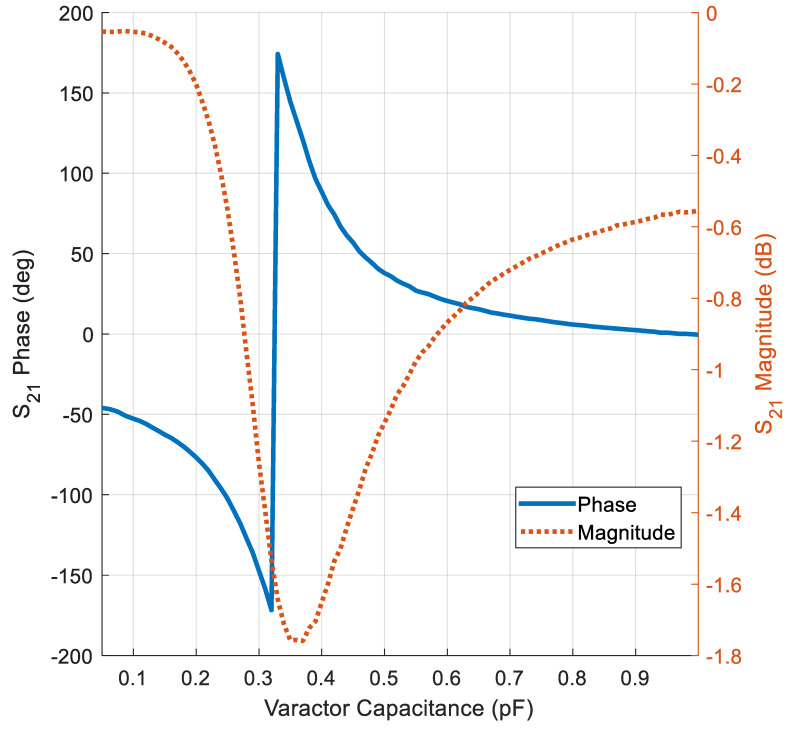
Behavior of the insertion loss and transmissive phase of the unit cell.

Furthermore, the phase variation covers 360° to the resolution of 45°, meeting the requirements for 3-bit phase modulation. The unit cell bandwidth is defined as the frequency range where the phase shift can achieve the desired phase variation while maintaining a transmission insertion loss below 3 dB [10,31]. With such a condition, the proposed transmissive unit cell achieves a bandwidth of 450 MHz, which spans from 9.75 GHz to 10.2 GHz, corresponding to a relative bandwidth of around 4.5%. At the center frequency, the performance of the unit cell is presented in Figure 5. The insertion loss is less than −1.65 dB while the phase is changing continuously with capacitor variation, showing the cell’s ability to design a TIS with high performance.

**Figure 6:**
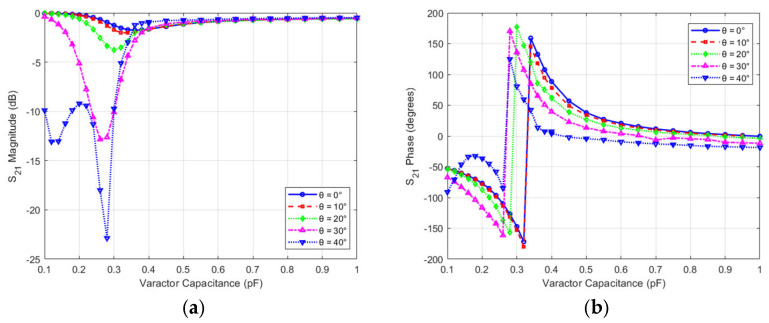
Effect of the incident angle and defocusing on unit cell performance with capacitance variation (**a**) Magnitude (**b**) Phase.

The performance of the unit cell may be affected by the incident angle of the electromagnetic waves. Therefore, the effect of an inclined incident plane wave on the amplitude and phase performance of the unit cell is studied for both variation in incident angle with the value of capacitance (i.e., which means variation of state) and also versus frequency. Figure 6a,b show the effect of theta variation versus capacitance on the phase and magnitude of the transmission coefficient at 10 GHz, respectively. The phase variations are almost tolerable (less than the state change phase, i.e., 45°) within a drift of 30° of incident angle, out of the normal incidence. However, the insertion loss or the transmission magnitude degraded severely with incident angles increasing of 20° for states that have capacitance values less than 0.36 pF and remained almost stable for other states. For example, State #6 has almost stable insertion loss, while the phase variation is significant for high incident angles.

**Figure 7:**
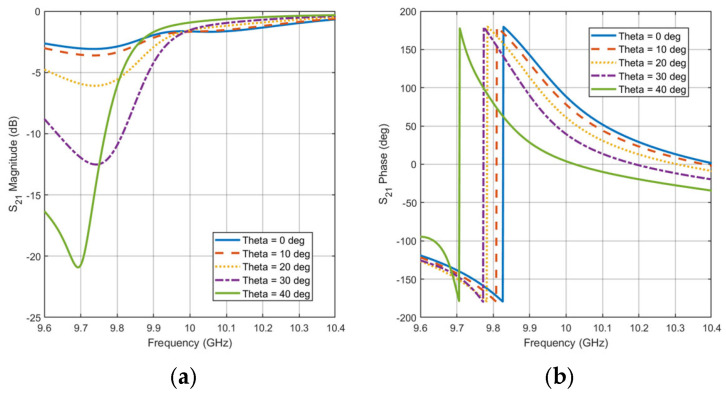
Effect of the incident angle and defocusing on unit cell performance with frequency variation (**a**) Magnitude (**b**) Phase.

Furthermore, the effect of the incident variation on the unit cell performance with constant state and frequency variation is studied. State #6 is selected to which has a low insertion loss at 10 GHz. Figure 7a,b show the effect of theta variation versus frequency on the phase and magnitude of the transmission coefficient, respectively. The phase difference is almost constant with frequency change within the bandwidth of the unit cell and has a tolerable change (i.e., less than 45°) within a drift of 30° of incident angle. The insertion loss is also affected at lower frequencies of the unit cell bandwidth and is still less than −2 dB at the center frequency, 10 GHz. Therefore, the designed transmissive unit cell is good for incident angles within 30°.

The performance of the proposed unit cell is compared with the literature, as shown in Table 2. The results show that eight reconfigurable states are accomplished to cover the 360°. This performance is achieved with low profile and insertion loss, making it candidate to design TIS for microwave application.

### 2.3. Design Method for the TIS Formation

**Figure 8:**
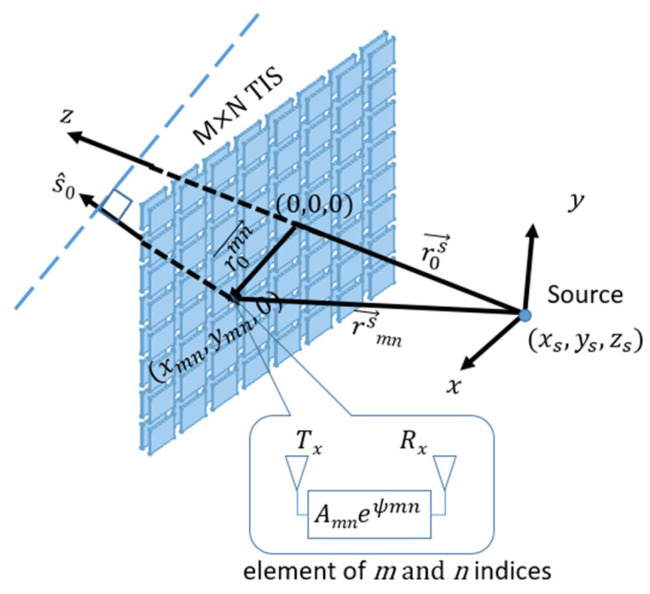
TIS configuration for beam steering to a specific direction.

The performance of TIS, designed for steering, depends on the ability of its individual unit cells to adjust their transmissive phases electronically. These phases can be programmed to be changed along the surface in various patterns, including linear, concentric, or angular distributions. This will enable specific electromagnetic wave behavior, such as beam focusing or beam steering. Therefore, through strategic unit cell design with tunable properties, a single TIS can do multiple functions. This study specifically examines a planner TIS employing concentric and angular phase gradient modes to manipulate spherical incident waves for beam focusing and steering applications. The incident waves leave the source in spherical waves and reach the TIS elements with different phases due to different path lengths. Similarly, they undergo different paths to reach the destination. By performing a phase correction for the scattered field at each element on the transmissive surface, incident waves from the feeding antenna will be adjusted to reach the destination with the same phase and so added constructively. Let us assume that a planar TIS is located on the *z*-plane with an odd number of elements M and N in the *x*-axis and *y*-axis directions, respectively. Even numbers of elements can also be considered with slight modification. The periodicity in the *x*-axis and *y*-axis are *d_x_* and *d_y,_* and the feeding source is located at a far-field region from it, as shown in Figure 8.

The location of each element, $\vec{r_{0}^{mn}}$, is the vector from the TIS center (0,0,0) to the center of each unit cell (*x_mn_*,*y_mn_*,0). It can be calculated as in Equation (1).

(1) $$x_{mn}=\left(m-\frac{M+1}{2}\right)d_{x}\;y_{mn}=\left(n-\frac{N+1}{2}\right)d_{y}$$

where *m* and *n* are the indices of the element in the *x*-axis and *y*-axis, respectively, *d_x_* and *d_y_* are the periodicity in *x* and *y* directions of the TIS, *M* and *N* are the size of the TIS in the *x*-axis and *y*-axis. For a feeding source (e.g., horn antenna) placed at location (*x_s_*, *y_s_*, *z_s_*), the radiated waves will propagate as spherical waves reaching TIS unit cells with different phases due to different paths between the source and each element. The length of the path from the source to the unit cell is the distance between center locations, which can be calculated using the magnitude (or norm) of the difference vector as in Equation (2),

(2) $$r_{mn}^{s}=\left\|r_{0}^{mn}-r_{0}^{s}\right\|=\sqrt{{\left(x_{mn}-x_{s}\right)}^{2}+{\left(y_{mn}-y_{s}\right)}^{2}+{\left(z_{s}\right)}^{2}}$$

Assuming we want to steer the beam to a specific direction of a unit vector $\hat{s}$, as in Figure 7. In this case, EM waves that leave the TIS should have the same phase in the direction plane, which is the normal plane to the unit vector of the steering direction. Therefore, phase compensation should consider the path difference in the incident waves from the source to TIS and the path difference in the EM waves from the unit cell to the direction plane. If the steering plane is parallel to the TIS plane, the departure path difference will be zero, which is the case for lens application. However, for a steering direction specified by (θ, φ), the unit vector of the normal plane in Cartesian coordinates is calculated as in Equation (3).

(3) $$\hat{s}=\left(\sin\theta \cos\varphi ,\;\sin\theta \sin\varphi ,\;\cos\theta \right)$$

The distance between the steering plane and the unit cell is the dot product of the unit vector, $\hat{s}$, and the unit cell position vector, $r_{0}^{mn}$ (look at Figure 7). To unify the phase of the dispersed field from the whole transmissive surface, the phase must be constant in a plane normal to the direction of the intended beam. For example, in the case of a transmissive surface with a feed whose phase center is at the origin, as shown in Figure 8, the unit cell phase compensation should satisfy the following:

(4) $$\psi_{mn}\;=\psi_{0}+{mod(k}_{0}\left(\hat{s}\cdot r_{0}^{mn}+r_{mn}^{s}-L_{0}\right),2\pi )$$

where *k*_0_ is the wave number that $k_{0}=c_{0}/\lambda$ where *c*_0_ is the speed of electromagnetic waves in free space, λ is the wavelength of the electromagnetic waves, and $\psi_{0}$ is the phase of the reference unit cell, which can be ignored.

### 2.4. TIS Results and Discussion

**Figure 9:**
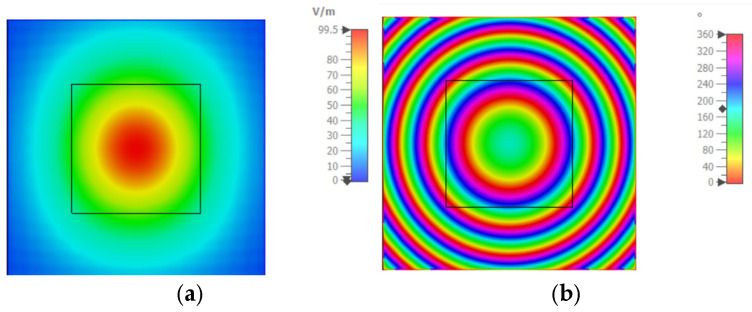
Electric Field distribution on z-plane (**a**) magnitude (**b**) phase.

This section validates the performance of the proposed unit cell in a full-scale TIS. A horn antenna is used as a feeding source for a full-scale planner TIS located at the *z*-plane. The first objective is to use TIS as a lens to focus the radiation pattern into a broadside direction. Firstly, the full-wave EM simulator (i.e., CST Microwave Studio) is used to simulate the horn antenna and estimate a suitable focal distance to place the TIS. The open boundary condition is set with an electric field monitor on a plane with dimensions of 432 mm × 432 mm at a distance of 200 mm away from the horn antenna to ensure enough distance for far-field results. The electric field magnitude and phase distributions on the field monitor plane at a distance of 200 mm are shown in Figure 9a and Figure 9b, respectively. Therefore, the horn antenna is located at z = −200 mm.

#### 2.4.1. TIS Configuration for Beam Focusing

**Figure 10:**
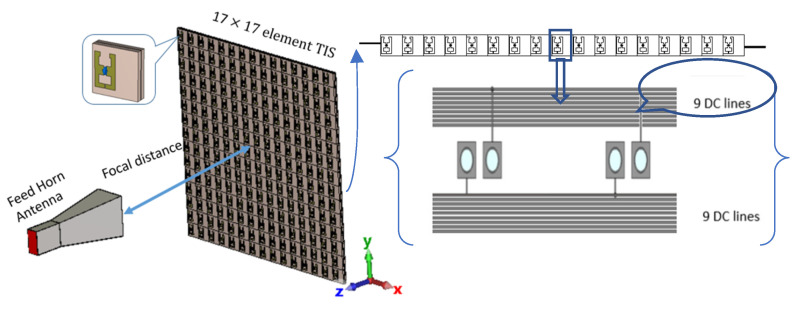
Full-scale simulation setup of 17 by 17 TIS.

**Figure 11:**
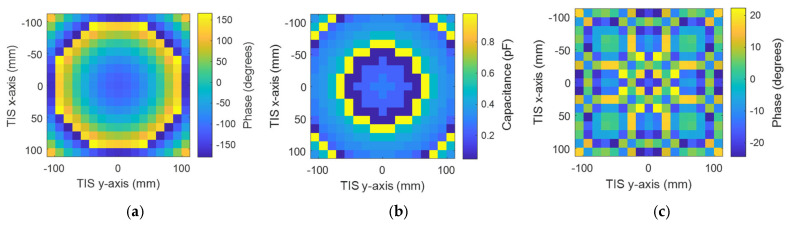
Phase configuration of 289-element TIS: (**a**) discrete phase, (**b**) required capacitance, and (**c**) phase discretization error.

The previously shown result of the field distribution corresponding to a studied plane, which is equivalent to M × N unit cells of 33 × 33 with periodicity in the *x*-axis and *y*-axis (i.e., *d_x_* and *d_y_*) of 13.2 mm. Though this plane confined almost the total electric field of the spherical waves in the broadside direction at this location, the simulation of such a size will need high computational resources or take a long time for limited resources, as in our case. Therefore, a TIS with (17 × 17) unit cells was built for simulation simplicity, which may confine most of the electric field intensity (look at Figure 8 for the black-line square). Therefore, a metasurface of 289 elements arranged in a planar array with (17 × 17) unit cells is designed for focusing the gain of the antenna into the broadside direction. Figure 10 shows the TIS structure and antenna built in a full-wave simulator, where the TIS is placed on the z-plane at a focal distance of 200 mm from the source antenna with matched polarization of antenna and unit cell elements. Each row has 18 lines of DC biasing, divided symmetrically along the y-axis as 9 lines up and 9 lines down. The outer network is symmetric around the x-axis since the upper nine lines will be on the left, and the lower nine lines will be on the right. One line is common or ground, while the other 17 lines connect the reverse bias voltage for each element as shown in Figure 10. Therefore, each element has its own lumped elements that are assigned the Varactors diode equivalent circuits, which can be controlled by assigning a suitable capacitance value. The results of the phase in Figure 8 show that the phase variation is smooth, while our TIS is built of discrete unit cells with periodicity of 13.2 mm. Therefore, the phase will be discretized to the size of the unit cell, which means that there will be discontinuity and discretization error. The unit cell phase required to focus the beam in the forward direction is calculated according to Equation (4). Then, the phase is quantized to the nearest state with minimal error. The calculated phase has a concentric distribution, as shown in Figure 11a, which is expected due to the spherical waves’ path difference. Using the phase response of the unit cell versus capacitance, the discrete phase is mapped into a suitable capacitance. The assigned capacitance is shown in Figure 11b, which is quantized to three-bit levels (eight states). The quantization error is shown in Figure 11c.

**Figure 12:**
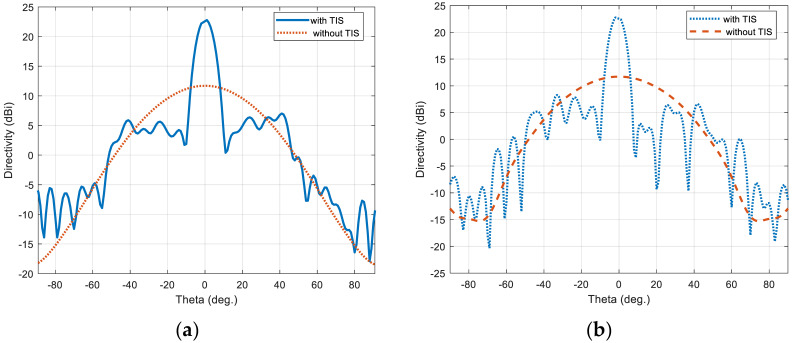
Directivity pattern of the horn antenna with/without TIS lens: (**a**) H-plane and (**b**) E-plane.

The full-wave simulator is used to evaluate the performance of the full-scale TIS when the elements are configured with the calculated required phase through the assigned suitable capacitance for each lumped element (i.e., varactor diode equivalent circuit). Figure 12a,b show the directivity patterns of the horn antenna with and without TIS in the H-plane and E-plane, respectively. The maximum broadside gain of 22.5 dBi is achieved with TIS, which means 10.8 dB improvement over the horn antenna without TIS. Moreover, the side lobe levels are acceptable for both H-plane and E-plane, which are around 16.3 dB of the worst side lobe located at around theta of 40°. The beam width of the main lobe is around 8.5° and 7.7° in the H-plane and E-plane, respectively.

**Figure 13:**
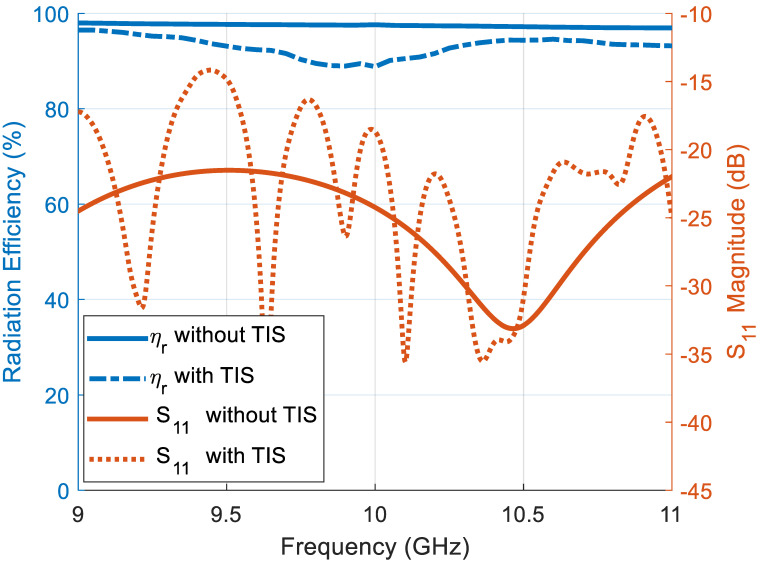
Antenna and TIS array radiation efficiency (on the left y-axis) and the reflection coefficient (on the right y-axis).

**Figure 14:**
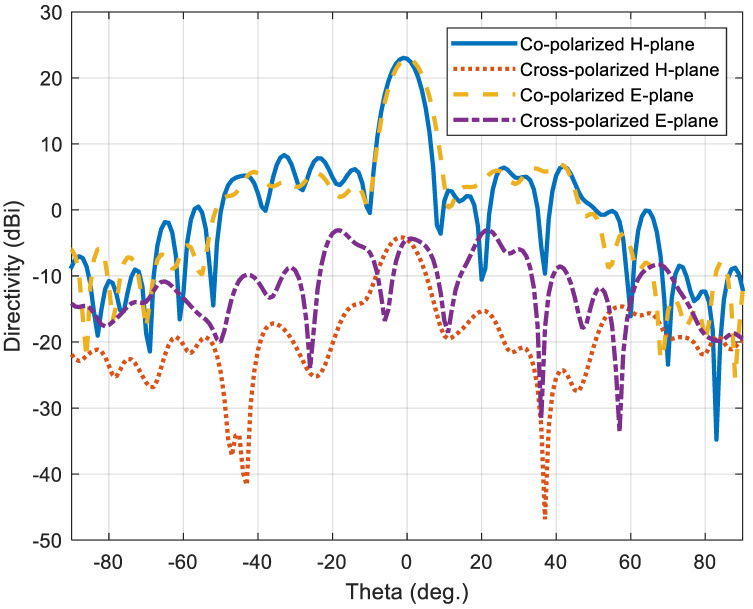
Polarization purity of the configured lens’ TIS illuminated by a pure polarized horn antenna.

On the other hand, polarization, the radiation efficiency (*η_r_*), and reflection coefficient may be affected by adding the TIS, consequently affecting the total performance of the antenna system. For this reason, the reflection coefficient at the antenna port and the radiation efficiency of the antenna before and after TIS addition are calculated using the full-wave simulator. Figure 13 shows these results, where the radiation efficiency scale is presented on the left *y*-axis while the reflection coefficient is on the right *y*-axis. The reflection coefficient is not affected significantly due to the far distance of the TIS position from the antenna. However, the radiation efficiency is reduced slightly, which is found to be 97.16% before TIS addition and 86.33% after addition at the center frequency, 10 GHz. This reduction is due to the added loss of the TIS lens structure. It is still acceptable with only 11% degradation, adding an insertion loss of −0.57 dB, which is very good. The polarization purity of the configured lens, illuminated by a pure polarized horn antenna, is presented in Figure 14 for both E-plane and H-plane. The cross polarization levels are −27.2 dB and −26 dB for E-plane and H-plane, respectively, which is better than [25].

**Figure 15:**
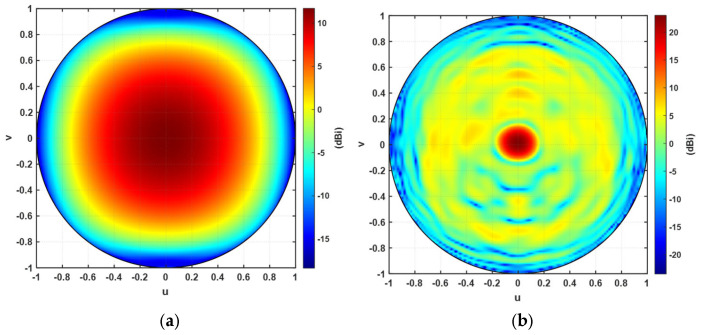
Orthographic projection of the antenna gain (**a**) without TIS lens and (**b**) with TIS lens.

The TIS lens is designed to focus and steer the beam into the forward direction. Therefore, the projection of the antenna pattern is a good way to present the improvement of the gain before and after the addition of the lens for comparison. Figure 15a,b show the orthographic projection of the antenna gain without and with the TIS lens, respectively. The results indicate a significant improvement in the gain at the broadside direction. Specifically, the scale of Figure 15b is larger than the scale of Figure 12a by around 10.8 dB. There is almost no significant side lobes except those of −16.3 dB, as also shown in Figure 12a,b. However, these side lobes are due to the introduced error of discretization and quantization of the phase and the mutual coupling effects. The characterization of the unit cell in the simulator considers periodic boundary conditions, which is an infinite array of the same unit cell. Therefore, the coupling is included, but it is assumed to have identical neighboring cells (i.e., an infinite array of the same element), which is not the case for focusing and steering applications where the unit cell has neighboring cells of different varactor biasing, resulting in different elements’ transmission coefficient phase and magnitude values compared with an ideal infinite array of identical elements. Moreover, the front phase of the wavefront is smooth circular rings, as in Figure 9, while the unit cell is square and has only eight states. This discretization and quantization will also affect the required smooth phase wavefront. Therefore, the presented loop-dipole unit cell was able to modify the phase of the configured TIS to reform the pattern of the antenna to achieve a 10.8 dB improvement. Another parameter used to compare the performance of a lens is the aperture efficiency (*η_a_*), which is defined as the effective area to the physical area of the structure, which is given by Equation (5).

(5) $$\eta_{a}=\frac{\lambda^{2}\eta_{r}D}{4\pi *A_{p}}\;$$

where λ is the wavelength of the EM wave, *η_r_* is the radiation efficiency, *D* is the directivity, and *A_p_* is the physical projection area of the structure. The calculated effective aperture of this configuration is 28.5% which is better than [2,24], showing the promising results of this TIS lens of low-profile structure.

**Figure 16:**
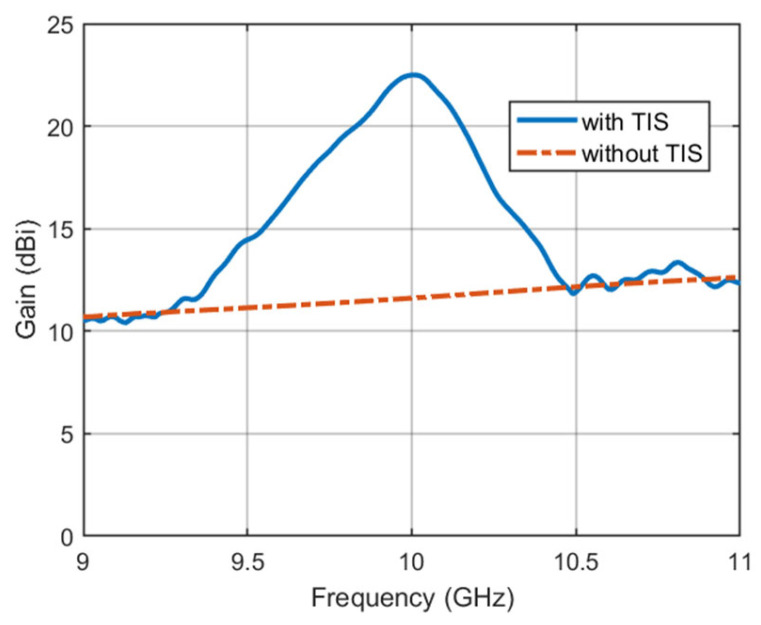
Antenna gain pattern versus frequency.

After improving the antenna radiation performance at the center frequency with and without TIS, it is important to study the effect of frequency on the configured TIS for focusing the beam. Figure 16 shows the antenna gain pattern versus frequency for the broadside direction. The performance of the TIS is significantly affected by the frequency. However, the improved performance is still acceptable for a low-profile structure. The bandwidth that the TIS lens has with this configuration is around 400 MHz, which is suitable for such a bandwidth application.

#### 2.4.2. TIS Configuration for Beam Steering

**Figure 17:**
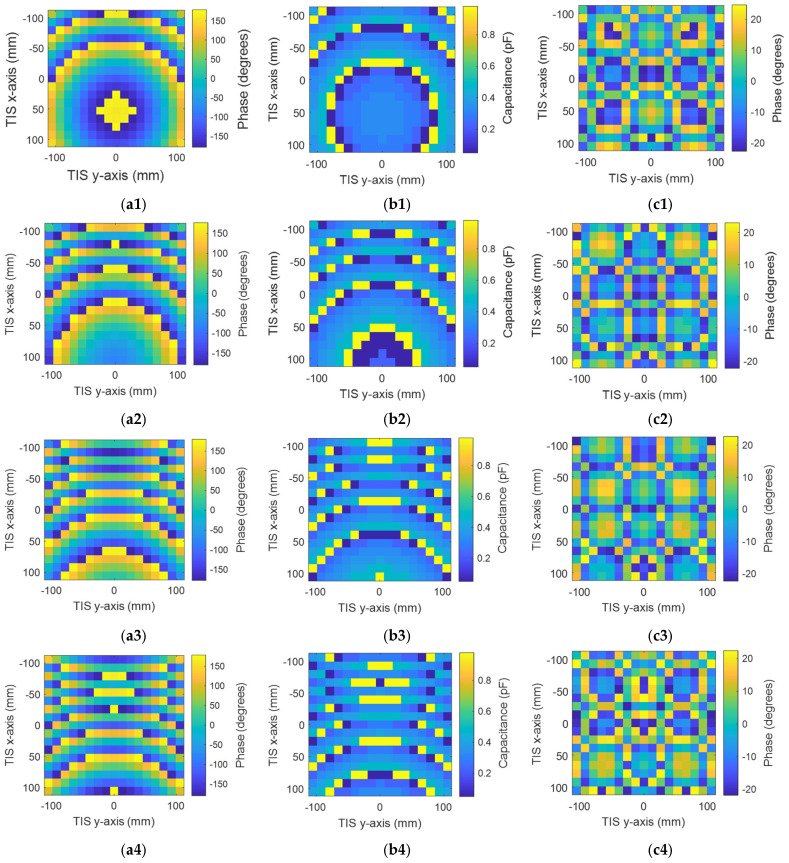
Configuration of the steering TIS to steer the beam to θ_s_ = (15°, 30°, 45°, 60°), respectively: (**a1**–**a4**) required phases, (**b1**–**b4**) assigned capacitances, and (**c1**–**c4**) phase quantization error.

For simplicity, we will use the same structure of antenna and TIS presented in the last section for the steering configuration. The steering will be studied for three scenarios: elevation direction, azimuthal, and a hybrid of both of them. It should be noted that all scenarios share the same concept of angular phase gradient distribution. Therefore, for the last two scenarios, only one steering angle (θ_s_) will be studied for simplicity. Firstly, the elevation direction is considered, where only four steering angles are studied as samples to save simulation time; θ_s_ = (15°, 30°, 45°, 60°). All the steering angles are positive (i.e., located in the upper half-space). However, the same idea can be applied to the second half. Theoretical phase calculation using Equation (4) is used to find the suitable phase distribution for each steering angle. Then, the phase is quantized and mapped to one of the eight phase cases through the assignment of varactor diode equivalent circuits with the corresponding capacitor values. Figure 16 shows the calculated phases, assigned capacitance, and phase quantization error for the four steering angles, respectively, starting from Figure 17a1–c1 for the first steering angle of theta = 15° to Figure 17a4–c4 for the fourth steering angle of theta = 60° in consequence. Results show the change in the angular phase distribution with the change in the steering angle. This phase makes the EM waves add constructively at the desired direction according to Equation (4).

**Figure 18:**
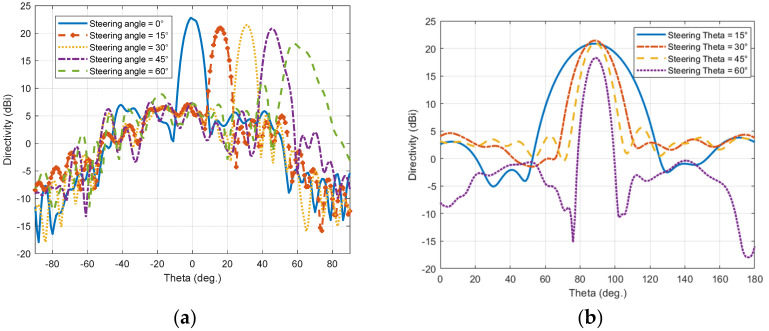
Steering pattern of the horn antenna with TIS: (**a**) E-plane and (**b**) H-plane.

The four configurations are simulated with the same structure and profile of the TIS lens presented in the last section to ensure validation of comparison and to imitate the real application. The only things to be changed are the values of the lumped elements’ capacitors, which are updated according to the desired steering angle. This update can be performed in a real application by switching the biasing voltage of the varactor diodes electronically. The resultant beams of these configurations are presented with the H-plane of the lens for comparison, as shown in Figure 18a,b for the H-plane and E-plate, respectively. Results show that the main beam of steered antenna radiation was found at θ_s_ = (16°, 31°, 46°, 58°). Comparing with the desired directions of θ_s_ = (15°, 30°, 45°, 60°), results show good accuracy in beam steering with a maximum difference of ±1° for the first steering cases (15°, 30°, 45°), with less than 2 dB reduction in maximum gain. However, this reduction increased to 4.5 dB at a steering angle of 60°, with −2° drift of steering angle (i.e., 58°). The steered beams, except for the steering angle of 60°, have side lobe levels around −14 dB, which is good for beam steering applications [6]. As the transmission phase profile is tilted to steer the beam in the H-plane, the projected aperture in the steering plane effectively decreases, leading to a broader beam there, while the orthogonal E-plane aperture remains unchanged and becomes more collimated under the stronger transverse phase gradient, resulting in a narrower beam as in Figure 18a,b.

**Figure 19:**
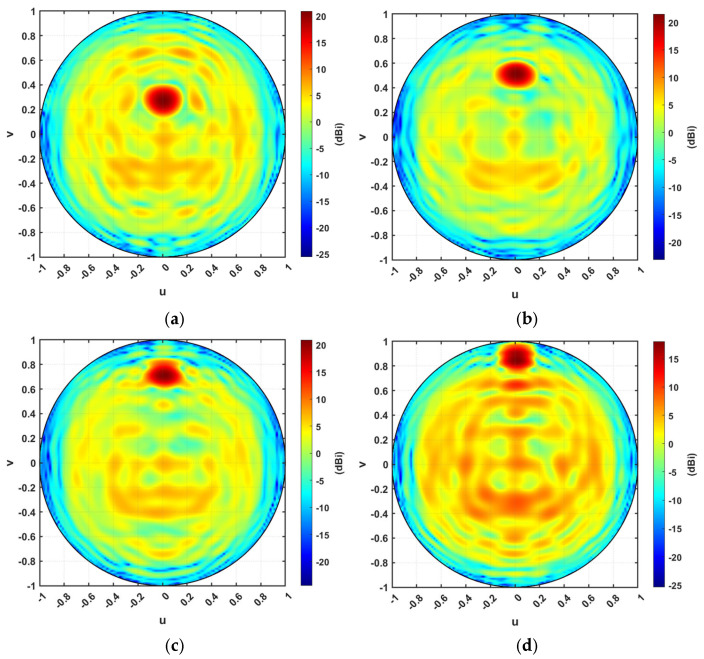
Orthographic projection gain pattern of the steering TIS at steering angles of (**a**) 15°, (**b**) 30°, (**c**) 45°, (**d**) 60°.

Figure 19a–d show the orthographic projection of the antenna directive pattern for the steering TIS at steering angles 15°, 30°, 45°, and 60°, respectively. It should be noticed that theta changes along the *v* vector while phi changes along the *u* vector. Therefore, as the steering angle increases, the pattern spot point becomes farther from the origin of the orthographic projection plot. The results indicate a significant improvement in the directivity at the desired direction, showing the ability of the proposed TIS to steer the beam with more than 10 dB gain over the source feeder. This steering can be controlled electronically through the configuration controller circuit.

**Figure 20:**
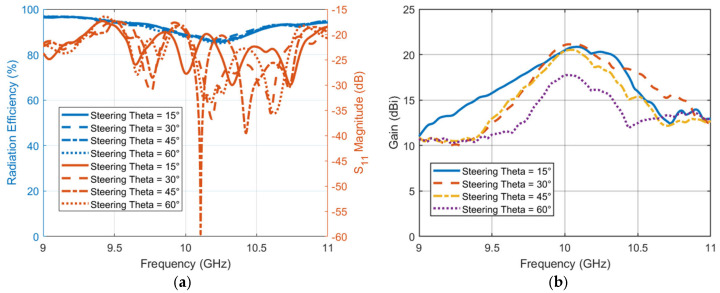
TIS performance versus frequency. (**a**) Antenna with TIS array reflection coefficient and radiation efficiency (**b**) gain.

The last step is to evaluate the performance of steering TIS with frequency for radiation efficiency, reflection coefficient, and directivity. A full-wave simulator is used to calculate the performance of the steering TIS at different frequencies. Figure 20a shows the radiation efficiency with scale on the left *y*-axis and the reflection coefficient scale on the right *y*-axis. The reflection coefficient is not affected significantly due to the far distance of the TIS position from the antenna (i.e., S_11_ < −17dB). However, the radiation efficiency is reduced slightly, with an average of around 87% at the center frequency, 10 GHz. This reduction is due to the added loss of the TIS structure. Moreover, the frequency of the reflection coefficient is almost the same as the source feeding antenna since TIS does not affect the bandwidth. However, it may be necessary to check the bandwidth within which the lens can have a directivity greater than a specific limit. Figure 20b shows the antenna gain pattern versus frequency for all the chosen steering angles or directions. The performance of the TIS is significantly affected by the frequency. However, the improved performance is still acceptable with such a low-profile structure. The average 3 dB gain loss bandwidth that the TIS lens has with this configuration is around 400 MHz. Therefore, the steering TIS is expected to be able to steer the beams electronically for many applications that require a gain of around 20 dBi. More gain can be obtained by extending the size of the TIS. Furthermore, the 15 dBi gain limit with this structure will have an average bandwidth of around 1 GHz.

**Figure 21:**
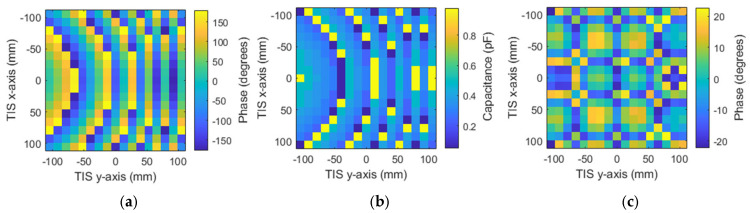
TIS configuration for azimuth steering of 45°: (**a**) discrete phase, (**b**) required capacitance, and (**c**) phase discretization error.

For the last two scenarios, the steering angles will be the azimuth angle of 45°, while the elevation is zero for the second scenario. In the third scenario, the hybrid direction is considered, where both azimuth and elevation angles are chosen to be 45°. Theoretical phase calculation using Equation (4) is used to find the suitable phase distribution for both scenarios. After that, the phase is quantized and mapped to one of the eight phase cases through the assignment of varactor diode equivalent circuits with the corresponding capacitor values. Figure 21a–c show the calculated phases, assigned capacitance, and phase quantization error of the azimuth steering angle of 45°, respectively.

**Figure 22:**
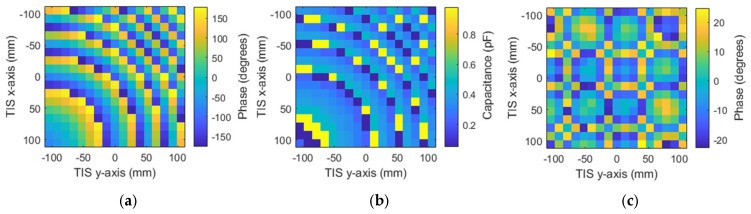
TIS configuration for azimuth and elevation steering of 45°: (**a**) discrete phase, (**b**) required capacitance, and (**c**) phase discretization error.

Similarly, Figure 22a–c show the calculated phases, assigned capacitance, and phase quantization error, respectively, of the hybrid steering direction of elevation angle 45° and azimuth angle of 45°. Results show the ability and accuracy of the configured TIS to steer the beam azimuthally or vertically.

**Figure 23:**
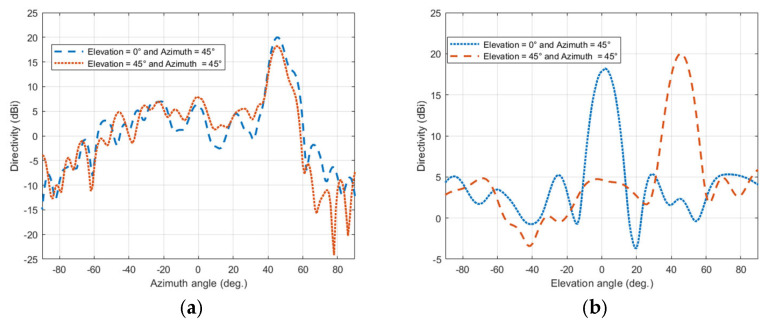
Steering pattern of the azimuth and azimuth elevation: (**a**) E-plane and (**b**) H-plane.

The resultant beams of the configurations are presented with the H-plane of the lens for comparison, as shown in Figure 23a,b for the H-plane and E-plane, respectively. Results show accurate beam steering with a maximum drift of ±2° from the value of 45°. The TIS was configured to steer the beam successfully in any direction with ±60° in both azimuth and elevation planes.

**Figure 24:**
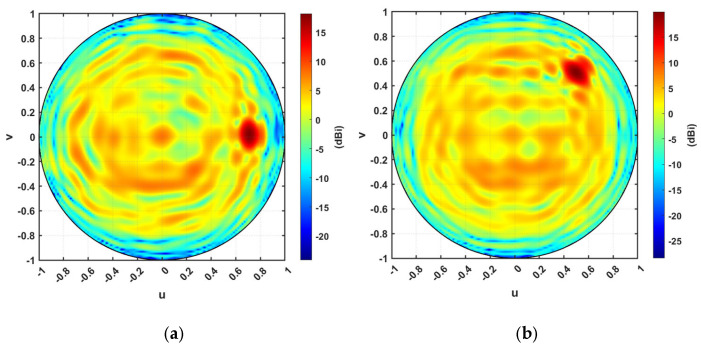
Orthographic projection gain pattern of the steering TIS at steering angles of (**a**) 15°, (**b**) 30°.

Figure 24a,b show the orthographic projection of the antenna directive pattern for the steering TIS at both azimuthal and azimuthal–elevation steering scenarios with a steering angle is 45°. The results indicate a significant improvement in the directivity at the desired direction, showing the ability of the proposed TIS to steer the beam for any direction with only reconfiguration, which can be performed electronically at fast switching time. This programmable steering will improve tracking and communication applications in 6G systems.

**Figure 25:**
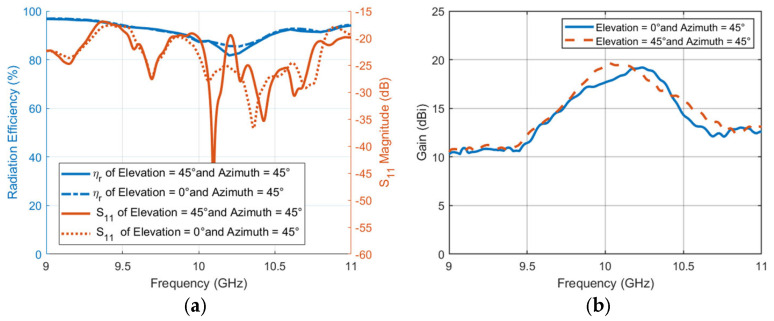
TIS performance versus frequency (**a**) reflection coefficient and radiation efficiency (**b**) gain.

The last step is to evaluate the performance of steering TIS with frequency for radiation efficiency, reflection coefficient, and directivity. For these scenarios, the reflection coefficient and radiation efficiency are on the same level as those of elevation-only steering, as shown in Figure 25a. The 3 dB gain loss bandwidth is almost the same as the previous, with an average of 400 MHz. However, there is degradation with around −2 dB on the maximum gain for azimuth steering, which may result from the effect of mutual coupling in the direction of polarization as shown in Figure 25b. This may be improved if the phase is optimized to account for mutual coupling, which is out of the scope of this article and may be considered in future research.

The performance of the configured TIS is compared with the literature, as shown in Table 3. The results show that the aperture efficiency of 28.5 is achieved at a high gain of 22.5 dBi. The performance of the designed TIS proves the ability of the loaded semi-loop dipole unit cell to control the phase of the incident wave without high insertion loss, which is more useful for narrow-band applications.

## 3. Conclusions

This paper presents loaded semi-loop dipole resonators to design a low-profile three-layer unit cell. The unit cell with a periodicity of 0.44λ at 10 GHz can modify the transmissive phase of the incident EM waves within 360° with a step of 45° by controlling the varactor diode with low insertion loss. The 17 × 17 array demonstrates a 10.8 dB gain enhancement for a horn antenna source at 10 GHz, with beam steering capability of up to 60° at ∓2° precision. The design achieves an average steering gain loss of 3 dB across a 400 MHz bandwidth, making it suitable for wireless coverage extension and tracking applications.

## Figures and Tables

**Table 1:** Unit Cell Dimensions in Millimeters.

Variable	Value (mm)	Variable	Value (mm)
L1	13.2	L7	1.5
L2	10.6	L8	2.35
L3	6.5	g1	2.7
L4	2.25	g2	0.8
L5	1.3	g3	0.2
L6	0.8	g4	0.4

**Table 2:** Unit cell performance comparison with the state-of-the-art.

Ref.	Frequency (GHz)	Reconfigurable Phase States	Periodicity	Bandwidth	Insertion Loss (dB)	Profile (Thickness)	Layers
[3]	5.2	7	0.58 λ_0_	1.35%	−2 *	0.156 λ_0_	6
[18]	3.6	6	0.72 λ_0_	2.7%	−4 *	0.095 λ_0_	5
[24]	24.125	8	0.28 λ_0_	NA	−2.75	0.323 λ_0_	5
[2]	12	NA	0.346 λ_0_	4% *	−3 *	0.126 λ_0_	3
This work	10	8	0.44 λ_0_	5%	−3	0.11 λ_0_	3

* Estimated from the graph.

**Table 3:** TIS performance comparison with the state of the art.

Ref.	Frequency (GHz)	Size	Profile (λ_0_)	SLL (dB)	Gain BW	AE (%)	Scanning Range (°)	Peak Gain (dBi)
[19]	29	14 × 14	0.125	−8 *	16.2%	15.9	60	19.8
[18]	3.6	6 × 6	0.095	−7	13.9%	NA	20	13.9
[24]	24.125	29 × 29	0.323	−9 *	5.1%	11.5%	30	19.85
[2]	12	22 × 22	0.126	−20.8	10.8%	17.0%	30	21
This work	10	17 × 17	0.11	−16.3	4%	28.5%	60	22.5

SLL: side lobe level, BW: bandwidth, AE: aperture efficiency, NA: not available, (*): estimated from the graph.

## Data Availability

The original contributions presented in this study are included in the article. Further inquiries can be directed to the corresponding authors.
